# Medical Societies

**Published:** 1879-09

**Authors:** 


### Editorial.

### MEDICAL SOCIETIES.

Is it wise to encourage the formation of medical societies with territorial limits including parts of two or more States, or the whole of a group of States? This question has been forced upon our attention several times during the last few years, by the formation and maintenance of the Inter-State Medical Society, embracing a small part of Illinois and Iowa, and a Tri-State Medical Society, embracing a large part of the States of Indiana, Illinois and Kentucky. And now, in looking over the proceedings of the Louisiana State Medical Society, we find that initial steps are being taken to organize a "Southwestern Medical Association," to embrace the States of Louisiana, Mississippi, Alabama, Texas and Arkansas. That the subject of a proper and efficient organization of the medical profession is one of great importance, will be admitted by all who have given it thoughtful attention. At the recent meeting of the American Medical Association, in Atlanta, Dr. S. E. Chaillé read a report or paper on the subject, in one of the Sections, that was deemed of sufficient interest to have the greater part of it read before the whole Association. In accordance with the recommendations of that paper, a committee of five was appointed, to report at the next annual meeting, on the ways and means for securing greater uniformity and strength of organization of the State Medical Societies and their auxiliary branches. At present a few of the State Medical Societies partake more of the nature of annual mass meetings of the profession than of permanently organized representative bodies, they having no affiliated local societies and receiving no regularly appointed delegates. This is more especially the case in Michigan.

The State societies in much the larger number of States are composed of a mixed membership, consisting in part of delegates appointed by district, county and city medical societies, and in part of permanent members who have either previously served as delegates or have been elected by direct vote of the society. Some of the State societies, and many of the local organizations, are incorporated by special legislative enactments, and many others are simple voluntary organizations, sustained only by such constitutions and by-laws as each has chosen to adopt.

If the mutual improvement of the members, and the direct advancement of medical science, were the only objects to be gained by medical society organizations, the diversities to which we have alluded would be of no importance. Neither would it be of any importance whether a society was composed of members living in parts of two or more States, or wholly in one State, or in the whole of three or four States. But this would be a very narrow and erroneous view of the objects that should be obtained, from a proper social organization of our profession. We have to deal not only with the acquisition of knowledge and its application in the treatment of disease, but we must deal equally with all the important questions involved in preventive medicine or sanitary science, as well as in the education and ethical relations of the profession itself. A proper view of these subjects will show any thoughtful mind that we have relations to the proper enlightenment of the public, and the guidance of legislation concerning many of the more important interests of general society, no less intimate or binding upon us than those pertaining to the direct treatment of the sick. The obligations arising from these relations to certain general interests of humanity, and to our own ethical and educational interests, cannot be efficiently performed without concert and concentration of action and expression; and this can be obtained in no other way than by professional organization and frequent interchange of views. To give force and efficiency to these mutual interchanges, and to expressions relating to questions concerning public health and welfare, the social organizations must be sufficiently complete to fully and fairly represent the whole profession; and in its subdivisions must correspond as nearly as possible with the municipal or legislative divisions of the people for whose interests we labor. If, as in most of the countries of Europe, all our laws were enacted and enforced by one general government, it would matter but little whether, in the formation of medical societies, any attention was paid to State or county lines or not. But with us, nearly all the legislation relating both to the education of our profession and the sanitary interests of the people, is done by State and municipal governments acting independently of each other. This fact alone is sufficient to show the necessity of having our professional organizations correspond mainly with these political or governmental divisions of the country. It is natural and proper that the physicians of each city, county and State should entertain a special interest in the sanitary condition and legislation of their own State or municipality, and should be better prepared to give the people advice concerning these matters than those of distant States. Again, only a small proportion of the whole number of practitioners can afford to leave home or go any distance to attend medical society meetings ; and only a very much smaller number can afford to leave home and spend a week to attend medical society meetings more than once or twice a year. From these considerations, it is obvious that in our country the natural plan for organization of the profession, and the only plan that admits of being made so complete as to include all the regular members of the profession, is that of city, county, state and national societies. The aim should be, to make the two first embrace in their membership all the regularly educated members of the profession in good standing, in their respective cities and counties ; and the two last should be essentially representative bodies—the state society receiving delegates on a uniform ratio of representation from the city and county societies, and the national organization receiving delegates in like manner from the state societies. This is essentially the plan now existing in a large proportion of the states. The city and county societies holding their meetings at the doors of their membership, can meet often and confer the benefits of free interchange of views and development of opinions on all the members, and as they elect the delegates to the state societies, these will concentrate the facts, opinions and policies developed in the local organizations, and give whatever is valuable a just and efficient influence in guiding public opinion and legislation in their respective States, concerning the sanitary interests of the people and the education of the profession ; and the national organization being composed essentially of delegates from the several state societies, should constitute a common bond of union for the whole, not only still further sifting and concentrating the facts, opinions and policies developed in the state societies, and through their several delegates returning them back revised and corrected, to become common property for the whole profession; but also by the free social intercourse of the delegates, more effectually destroying local prejudices and sectional rivalries, and uniting the profession of all the states in fraternal fellowship. It is easy to see that if such a plan was actually carried into effect in each state and territory, it would constitute so complete an organization that it would be possible for our profession to regulate all matters pertaining to the education and qualifications of its members, as the clergy do through their conferences, synods and diocesan assemblies — or the legal profession through the courts. Is it not, then, greatly to be desired that all possible influence shall be concentrated in the direction of extending and perfecting the local social organizations of the profession, and in making the state and national organizations more strictly representative in their character, more efficient in their modes of work, and more definite in their plans and objects? Will not the organization of societies intermediate between the State and national associations necessarily tend to divert interest and attention from both of these ? If we form a Southwestern Medical Association, why not a southeastern, a northwestern and a northeastern as well ? If we have a *Tri-state* society in one instance, why not organize bi-state, tri-state or quadruple-state societies all over the Union ? But if such organizations multiply, is there not danger that both the individual state, and the national associations, will be so far weakened and disintegrated as to deprive them of all efficiency, and the profession find itself again fostering mere sectional interests and prejudices, instead of State efficiency and national harmony. The subject is one of great intrinsic importance, and we hope it may receive the attention it deserves.

